# Omics Biomarkers in Obesity: Novel Etiological Insights and Targets for Precision Prevention

**DOI:** 10.1007/s13679-020-00393-y

**Published:** 2020-06-27

**Authors:** Krasimira Aleksandrova, Caue Egea Rodrigues, Anna Floegel, Wolfgang Ahrens

**Affiliations:** 1 Nutrition, Immunity and Metabolism Senior Scientist Group, Department of Nutrition and Gerontology, German Institute of Human Nutrition Potsdam-Rehbruecke (DIfE), Nuthetal, Germany; 2 Institute of Nutritional Science, University of Potsdam, Potsdam, Germany; 3 Department of Epidemiological Methods and Etiological Research, Leibniz Institute for Prevention Research and Epidemiology–BIPS, Bremen, Germany; 4 Faculty of Mathematics and Computer Science, University of Bremen, Bremen, Germany

**Keywords:** Obesity, Biomarkers, Genomics, Transcriptomics, Proteomics, Metabolomics

## Abstract

**Purpose of Review:**

Omics-based technologies were suggested to provide an advanced understanding of obesity etiology and its metabolic consequences. This review highlights the recent developments in “omics”-based research aimed to identify obesity-related biomarkers.

**Recent Findings:**

Recent advances in obesity and metabolism research increasingly rely on new technologies to identify mechanisms in the development of obesity using various “omics” platforms. Genetic and epigenetic biomarkers that translate into changes in transcriptome, proteome, and metabolome could serve as targets for obesity prevention. Despite a number of promising candidate biomarkers, there is an increased demand for larger prospective cohort studies to validate findings and determine biomarker reproducibility before they can find applications in primary care and public health.

**Summary:**

“Omics” biomarkers have advanced our knowledge on the etiology of obesity and its links with chronic diseases. They bring substantial promise in identifying effective public health strategies that pave the way towards patient stratification and precision prevention.

## Introduction

The obesity pandemic has emerged as a leading global public health threat of the twenty-first century increasingly spreading across both developed and developing countries with the most vulnerable and socially disadvantaged population groups being most affected [1, 2]. Despite the growing recognition of the problem, obesity prevalence has nearly tripled since 1975 affecting billions of people around the world. According to estimates of the World Health Organization (WHO), in 2016, 1.9 billion people (40% of the world population) were overweight, and of these, over 650 million (13% of the world population) were obese [3]. There is a worrying tendency of climbing rates of morbid obesity, especially among children [4].

Long considered a merely intermediate chronic disease “risk factor” or socially unacceptable behavior reflecting a lack of willpower, obesity was recently recognized as a systemic chronic disease related to excessive and abnormal accumulation of body fat leading to adverse health effects. Obesity was defined as a multi-causal chronic disease recognized across the life span resulting from long-term positive energy balance with the development of excess adiposity that over time leads to structural abnormalities, physiological derangements, and functional impairments [5]. Obesity increases the risk of developing numerous comorbidities (e.g., type 2 diabetes, non-alcoholic fatty liver disease, cardiovascular disease, and certain types of cancer) and increased premature mortality [6]. Despite its rapidly increasing prevalence across the globe, obesity as a public health threat has not yet received the same urgent attention as it has rapidly spreading infectious diseases. Until today, public health initiatives have not been able to reverse the accumulating burden of obesity in any population [7]. So far, there is little evidence of successful population-level intervention strategies that reduce the high prevalence of obesity in populations across the globe effectively. With most recently published statistics on the alarming magnitude of the pandemic, the urgency of better strategies for preventing and management of obesity has never been more obvious. The pathogenesis of obesity is far more complex than just an imbalance between energy intake and expenditure leading to passive accumulation of excess weight. Recent research has highlighted the importance of the gene-environment interactions (epigenetic modifications), and complex and persistent hormonal, metabolic, neurochemical, and immune-inflammatory disturbances involved in obesity development [8]. To date, the specific regulatory alterations and metabolic consequences of excess and prolonged accumulation of body fat are not clearly understood.

**Fig. 1:**
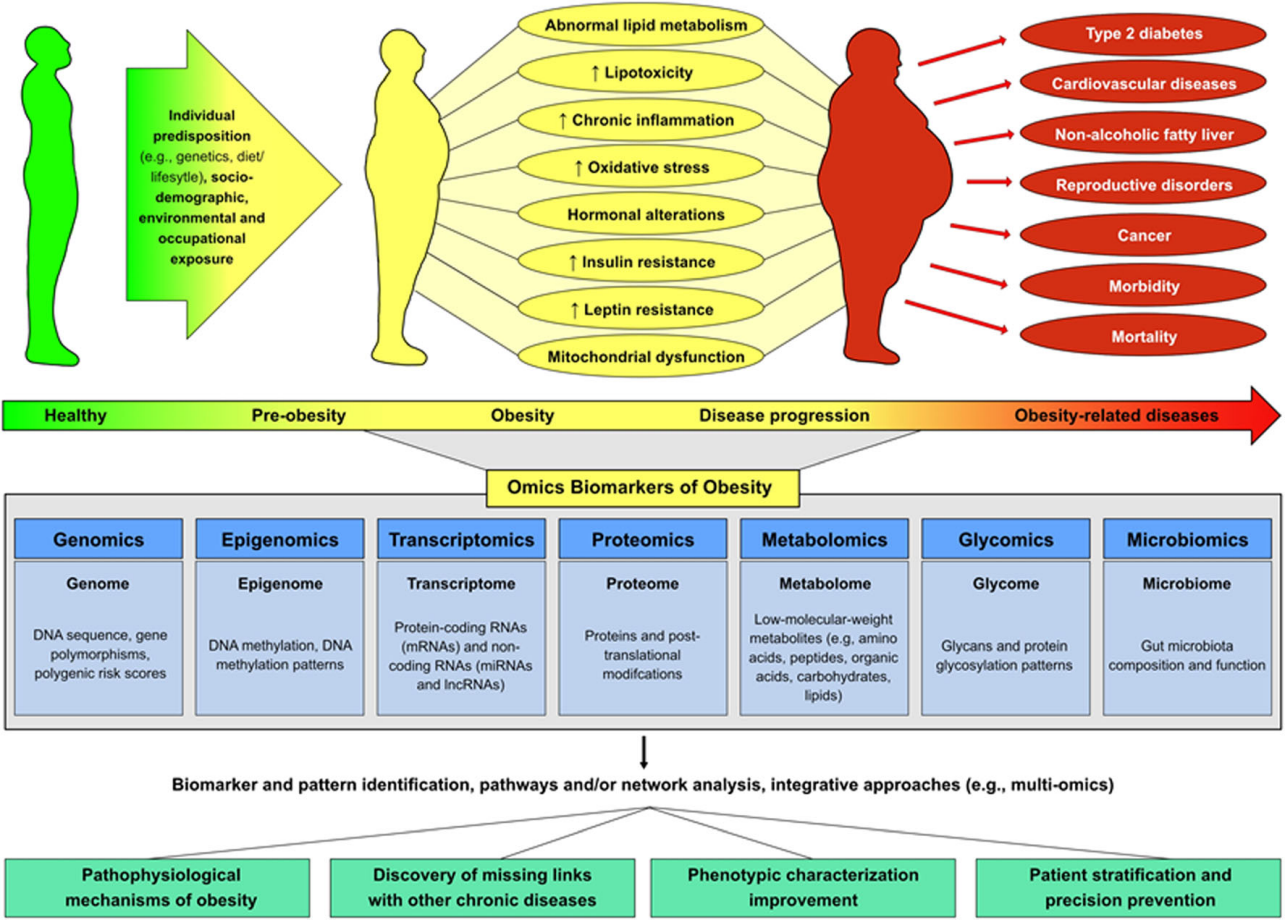
“Omics” platforms in obesity research

Technology advancements during the last decades and the paralleled “omics” revolution have brought forward an accelerated research incentive and a new promise for an improved understanding of the mechanisms explaining the complex biology behind obesity [9]. The identification of novel “omics” biomarkers could bring forward the knowledge on the etiology of obesity and its pathophysiological links with chronic diseases [10]. Furthermore, “omics” biomarkers could aid in getting a refined characterization of obesity phenotypes and serve as targets for precision prevention and therapy [9, 11]. Intensified efforts in “omics” research have been invested in the identification of genes (genomics), messenger RNA (mRNA) and microRNAs (miRNAs) (transcriptomics), proteins (proteomics), and metabolites (metabolomics) [12]. Other “omics” platforms that provide insights into the regulation of biological pathways include epigenetic markers—mostly DNA methylation—of gene expression and phenotype (epigenomics) and gut microbiota (microbiomics). Different analytical platforms and bioinformatic tools have been developed to explore the abundance of generated data such as individual omics-based approaches, pathways, and/or network analyses. Newer trends include integrative approaches such as multi-omics and trans-omics analyses [13] (Fig. 1*.* “Omics” platforms in obesity research).

This narrative review presents an overview of the development and recent highlights in published research on “omics” biomarkers in obesity from human epidemiological studies in understanding the etiology and pathophysiology of obesity and its phenotypic characterization.

## Search Strategy and Selection Criteria

References were identified by searches of PubMed with the terms “obesity,” “adiposity,” “central obesity,” “body mass index,” “waist circumference,” “waist-to-hip ratio,” “BMI,” “WHR,” “body fat,” “fat mass” in combination with the terms “omics,” “biomarkers,” “genome,” “genomics,” “genome wide association study,” “GWAS,” “epigenome,” “epigenomics,” “transcriptome,” “transcriptomics,” “miRNA,” “metabolomics,” “proteomics,” “lipidomics,” “glycome,” “glycomics,” “microbiome,” and “microbiomics.” Bibliography lists of the identified publications were also screened to identify additional articles. The search mainly focused on papers published from January 1, 2015 to March 31, 2020; however, previous review articles and key publications that shaped the field were also included.

## Genomics

Evidence on genetic origins of obesity emerged in the 1970s with twin and adoption studies, providing first clues on genetic heritability of obesity that has been estimated to be 40 to 70% [14]. In the 1990s, the discovery of leptin and leptin receptor genes and the leptin-driven melanocortin 4 signaling pathways prompted a sequence of genetic studies that uncovered *rare* mutations in single genes regulating appetite leading to early-onset extreme obesity [15]. This rare monogenic form of obesity is largely caused by high-risk genetic variations involved in the control of appetite and energy maintenance along the leptin-melanocortin pathway [15]. A number of variants involved in monogenic obesity have been described in the literature [15, 16]. However, for the majority of the population, obesity is multifactorial and genetic susceptibility is determined by the influence of multiple genetic variants [16, 17]. Genomic research has rapidly developed in the last two decades due to the development of DNA-microarrays-based techniques and next-generation sequencing (NGS) that allow mapping of the generated sequences and analyses of population-specific genetic traits [18]. With the emergence of genome-wide association studies (GWASs), hundreds of genetic variants involved in different biological pathways (e.g., central nervous system control of food intake and energy expenditure, food sensing and digestion, adipocyte differentiation, and insulin signaling) have been associated with polygenic obesity [19–21]. A recent GWAS based on 700,000 individuals identified 941 near-independent single-nucleotide polymorphisms (SNPs) associated with BMI [22]. Among specific genes explored in the different GWASs, the *FTO* and *MC4R* genes have emerged as major contributors to all polygenic obesity phenotypes [17, 23]. Notably, the genes at different loci seem to work in interaction with each other converging on certain pathways and networks differentially reflecting biological processes associated with fat accumulation and fat distribution [24]. Despite the enormous number of discovered loci, these collectively explained less than 3% of the variance of BMI observed [22]. Part of the gap between the explained genetic variance of BMI and the estimated heritability (40–70%) could be accounted for by the inability of GWASs to detect loci that are associated with traits whose effect sizes are too small to reach genome-wide statistical significance. To address this aspect, a number of studies attempted to develop effective multi-locus profiles of genetic risk for obesity, known as genetic risk scores (GRS). However, these scores only showed a relatively low correlation with measured BMI ranging from 0.01 to 0.12 [17]. Obtaining meaningful predictive power of a polygenic score is dependent on the information from multiple common variants. As compared to scores that were based on a restricted number of loci, a new GRS has been generated to predict BMI based on 2.1 million common genetic variants measured in 306,135 individuals [25]. This GRS outperformed previous scores from the GWAS that reached genome-wide statistical significance in predicting obesity and weight gain [25]. For instance, the 2.1 million-variant score showed a stronger correlation with BMI (correlation coefficient 0.29) as compared with the 141-variant score (correlation coefficient 0.13) [25]. Larger discovery GWASs and computational algorithms would likely lead to an increased ability of future GRS to identify high-risk individuals and could facilitate targeted strategies for obesity prevention that can start already in early childhood. Furthermore, since individuals at elevated genetic risk are also most susceptible to risk posed by obesogenic environments, GRS could be helpful in guiding lifestyle interventions targeted at high-risk individuals [26].

## Epigenomics

Recent years witnessed an unprecedented boost of research on understanding the role of the human epigenome in health and disease. Epigenetic regulation can involve DNA modifications (e.g., DNA methylation), histone modifications, and non-coding RNAs (e.g., miRNAs), and may affect all DNA-based processes without altering the DNA sequence [27]. Epigenomic biomarkers, mostly defined based on DNA methylation of cytosines in cytosine-guanine dinucleotides (CpG), are prone to changes in response to environmental factors and could reflect different developmental windows over the human life span. Such biomarkers can be determined based on whole-genome bisulfite sequencing and epigenomic array-based technologies [28]. The importance of epigenetic changes has been first acknowledged by human epidemiological studies that provided evidence that prenatal and early postnatal environmental factors influence behavioral disorders and exert increased chronic disease risk later in life [29, 30]. In particular, early-life exposures to stress, under- or overnutrition during gestation or lactation, are associated with overweight or obesity in later adulthood [31]. Epigenetic dysregulation through DNA methylation of genes involved in growth, inflammation, lipid metabolism, glycolysis, or adipogenesis may explain these associations [32, 33]. Data from the Dutch Hunger Winter Families study suggested significant differences in DNA methylation patterns associated with periconceptional famine exposure [34, 35]. Recently, epigenome-wide association studies (EWASs) provided new lines of evidence on the association between genome-wide (array-based) DNA methylation and obesity or related phenotypes [36–39]. For example, a recent EWAS using whole-blood samples from 5387 individuals from the EPICOR, KORA, and LOLIPOP cohorts identified changes in DNA methylation of 187 genetic loci associated with BMI. Gene set enrichment analysis revealed that altered patterns were observed in genes involved in lipid and lipoprotein metabolism, substrate transport, and inflammatory pathways [36]. In another EWAS study based on a sample of 641 participants in the REGICOR study and a validation sample of 2515 participants in the Framingham Offspring cohort, 70 CpG regions were associated with BMI and 33 CpG regions [37]. These markers explained ~ 26% and ~ 29% of the variability of BMI and waist circumference, respectively [37]. Of note, very few methylation pattern variations of the CpG regions were consistently associated with obesity across published studies, which may indicate a large number of false-positive findings in EWAS studies [40]. Due to methodological challenges, human studies on histone modifications in obesity have been sparse. Especially, *omics*-based studies have been lacking. So far, one study used the chromatin immunoprecipitation (ChIP) method to analyze histone methylations in adipose tissue of a cohort of 39 patients with different metabolic profiles [41]. The results suggested that H3K4me3 enrichment in the promoter of several factors involved in adipogenesis, lipid metabolism, and inflammation in visceral adipose tissue, i.e., LEP, PPARG, IL6, and TNF, were directly associated with higher BMI and metabolic deterioration [41]. Observations like these support the role of epigenetics in obesity risk. Further epigenomic analyses using novel “omics” techniques, such as CHIP sequencing, would be warranted to evaluate histone modification biomarkers in human obesity research.

Overall, epigenetic changes are plastic and dynamic, and finding out if they are a cause or a consequence of disease has been challenging. Methylation patterns and histone modifications vary across different cell types and over time [42] and can be influenced by multiple extrinsic and intrinsic factors, making epidemiological data difficult to interpret [43].

## Transcriptomics

Transcriptomics may bridge the gap between GWAS and physiological studies by deciphering information residing in genes [44]. Transcriptomic biomarkers include protein-coding RNAs (mRNAs) and non-coding RNAs (ncRNAs) that can be measured using RNA sequencing and array-based gene expression methods [45]. Tissue-specific analyses of the mRNA transcriptome of adipocytes from visceral and subcutaneous fat cells revealed more than a thousand genes whose expression was altered in obese as compared to lean individuals [46, 47]. Due to the rare availability of tissue samples in large epidemiological studies, alteration in the peripheral blood transcriptome was used as a valid alternative in the identification of transcriptomic biomarkers in obesity [47]. Whole-blood mRNA levels determined by array-based transcriptional profiling were correlated with BMI in two large independent population-based cohort studies (KORA F4 and SHIP-TREND) comprising a total of 1977 individuals [46]. The obesity-associated gene expression signatures pointed to key metabolic pathways involved in protein synthesis, enhanced cell death from proinflammatory or lipotoxic stimuli, enhanced insulin signaling, and reduced defense control against reactive oxygen species [46]. Protein-coding genes represent less than 2% of the total genomic sequence, whereas about 98% of the DNAs are transcribed as ncRNAs [48]. The development of high-throughput sequencing technologies allowed the identification of ncRNAs, such as miRNAs and long ncRNAs (lncRNAs) [48]. miRNAs elicit post-transcriptional repression of gene expression and several studies suggested that specific miRNAs were differentially expressed in adipose tissue of obese individuals as compared to those with normal weight [49]. miRNAs have shown to exert important regulatory roles in adipogenesis, adipocyte differentiation, and insulin signaling [50, 51]. Although these findings require invasive methods for sample collection (biopsies of adipose tissue) and consequently are based on an only a limited number of participants—often from clinical studies—they provide valuable insights into the mechanistic understanding of the ongoing progressive disbalances observed during obesity progression [52, 53]. On the other hand, circulating miRNAs (cmiRNAs) are released by tissues into the bloodstream and, therefore, are regarded as promising candidate biomarkers for further clinical application since samples can be collected by minimally invasive methods [44]. As cmiRNAs are released into the bloodstream, they serve as key messengers between cells and tissues, participating in the metabolic organ crosstalk [54]. A recent systematic review identified 33 cmiRNAs with dysregulated expression in serum or plasma in people with obesity compared to lean controls that have been replicated by two or more independent research groups [55]. A majority of the genes identified via obesity-related cmiRNAs is involved in fatty acid metabolism and phosphoinositide 3-kinase (PI3K-Akt) pathways [55]. In addition to the miRNAs, recently, lncRNAs also gained importance in obesity research as key regulators of adipogenesis, inflammation, and insulin sensitivity [56–60]. For example, a functional lncRNA arising from the CEBPα locus involved in adipogenesis was shown to prevent CEBPα gene methylation, resulting in elevated expression of the CEBPα mRNA [61]. Overall, transcriptomic studies face innumerous challenges, including the fact that the transcriptome varies by tissues and cell types as well as within these tissues and over time. Although an exciting prospect, the isolation and profiling of cmiRNAs from human samples remains challenging, mostly due to their extremely low concentrations. Differences in sample extraction, cmiRNA isolation, quantification, or profiling methods may yield inaccurate and/or non-reproducible results [62]. More research on ncRNAs that integrates experimental and bioinformatic tools is needed to gain a better knowledge of whether they could be successfully applied in preventive and clinical care.

## Proteomics

Proteomics has emerged as a powerful tool in the identification and biochemical characterization of proteins that are associated with obesity and its comorbidities. It has the advantage of being capable of detecting protein post-translational modifications and protein interactions that cannot be detected by genomics and transcriptomics. The most common bioanalytical platforms for proteomic analysis include matrix-assisted laser desorption/ionization-coupled with time-of-flight mass spectrometry (MALDI-TOF-MS), liquid chromatography coupled with electrospray ionization mass spectrometry (LC-ESI-MS), surface-enhanced laser desorption/ionization mass spectrometry (SELDI-TOF-MS), and protein microarray [63]. Secreted proteins constitute an important class of molecules expressed by approximately 10% of the human genome [64]; therefore, serum/plasma proteome provides a useful resource for monitoring molecular events of pathophysiological changes that occur in obesity [63]. Most population-based proteomic studies in obesity have been based on small samples and had limited analytical and outcome reproducibility [65–69]. A recent study characterized and compared the plasma proteomes of two large independent cohorts of obese patients in Canada and Europe including 1002 obese and overweight individuals using shotgun MS-based proteomic measurements. Statistically significant associations with BMI could be seen for the following biomarkers: complement factor B (CFAB), complement factor H (CFAH), complement factor I (CFAI), C-reactive protein (CRP), proline-rich acidic protein 1 (PRAP1), and the calprotectin complex formed by proteins S100-A8 and S100-A9 [70]. Among these proteins, CRP showed the strongest association with BMI and it was also associated with all identified biomarkers. Altogether, these findings suggest that chronic inflammation in obese persons could represent the underlying reason for the associations of these biomarkers with obesity [70]. Further well-designed epidemiological studies based on proteomic analyses are warranted to determine signature proteins that can serve as biomarkers for obesity and related diseases.

## Metabolomics

Metabolomics pursues to measure the totality of metabolites in a given biological system [71]. Metabolites represent a diverse group of low-molecular-weight structures among them lipids, amino acids, peptides, organic acids, and carbohydrates. Most recently, lipidomics emerged as an important branch of metabolomics with special relevance to obesity research [72]. There are two analytical approaches, untargeted and targeted metabolomics which can be based on nuclear magnetic resonance (NMR) spectroscopy or MS technologies. On the one hand, untargeted metabolomics uses an exploratory design and simultaneously measures up to several thousand metabolites, but many of them may remain unidentified. On the other hand, targeted metabolomics is limited to a predefined set of metabolites but provides metabolite identity and often quantitative data. Both strategies found distinct metabolic alterations in obese compared to lean subjects across different study populations, including higher plasma levels of branched-chain amino acids (BCAA) and aromatic amino acids and lower plasma levels of glycine, as well as higher plasma levels of acylcarnitines, fatty acids, and certain phospholipids [73–76]. Higher concentrations of BCAA and aromatic amino acids and lower concentrations of glycine have also been linked to insulin resistance [77] and a higher risk of type 2 diabetes [78, 79]. Increased levels of BCAA were also suggested to enhance activation of the mammalian target of rapamycin (*mTOR*) signaling, oxidative stress, mitochondrial dysfunction, and apoptosis [80]. Via these pathways, BCAA may be involved in the pathophysiology of obesity and associated diseases and may therefore serve as a promising target biomarker. Further mechanistic and epidemiological studies are needed to understand the role of BCAA in these chronic diseases and might lead to the recommendation to limit BCAA intake (e.g., to those at higher risk of developing these chronic diseases) , and the establishment of therapeutic intervention that might ameliorate BCAA-driven dysregulation in cellular signaling and consequent maladaptive phenotypes [80]. Overall, metabolomics has a high potential to improve precision medicine of serious metabolic diseases such as obesity through a more precise patient stratification and monitoring and might lead to the development of intervention strategies, including drug discovery and testing [81].

## Lipidomics

Lipidomics is a branch of metabolomics that is focused on measuring lipid species in a given biological system [72]. Divided into fatty acyls, glycerolipids, glycerophospholipids, sphingolipids, sterols, and prenols [82], the large chemical diversity of these molecules and their dynamic change in response to physiological and environmental factors represent challenges for their analytical determination and quantification and for understanding their biological roles. A combination of different bioanalytical techniques is necessary to achieve high sensitivity and high specificity of complex untargeted and targeted lipidomic experiments [83, 84]. For decades, simple lipid profile analysis has been a fundamental tool in clinical practice to assess dyslipidemia [85]. Technological advancements in MS allowed understanding that numerous other plasma lipids are mediators of metabolic dysfunction and disease progression in obesity and obesity-related chronic diseases, transcending the clinical commonly used lipid panel [86]. Previous lipidomic studies identified higher concentrations of short- and medium-chain acylcarnitines in obese compared to lean subjects which may result from impaired fatty acid biosynthesis and oxidation [87]. In addition, plasma concentrations of free fatty acids, in particular, proinflammatory omega-6-fatty acids, were shown to be increased in obesity as a result of stress to adipose tissue [75]. Using targeted metabolomics in the EPIC-Potsdam study, we previously found that mostly phospholipids from the diacyl-phosphatidylcholine subclass were positively correlated with BMI and waist circumference, whereas acyl-alkyl-phosphatidylcholines and some lysophosphatidylcholines were negatively correlated with these obesity measures [74]. Other studies confirmed distinct phospholipid profiles in obesity [73, 76]. Phosphatidylcholines are primarily synthesized hepatically and secreted as part of blood lipoproteins [88]. We previously showed in the EPIC-Potsdam study that diacyl-phosphatidylcholines were positively correlated with triglyceride concentrations, whereas acyl-alkyl-phosphatidylcholines were positively correlated with high-density lipoprotein cholesterol [79]. Thus, they may represent a more complex picture of dyslipidemia in obesity. In addition, an intervention study suggests that high-fiber diets may beneficially alter metabolic profiles of phospholipids in obese individuals [89]. This study provides one example of the application of lipidomics in the development of obesity prevention strategies.

## Glycomics

Glycans are ubiquitously present in all cells. They are essential for cellular physiological processes and are linked to other biomolecules such as lipids (glycolipids) and proteins (glycoproteins and proteoglycans) through glycosidic linkages to form glycoconjugates [90]. Around 70% of the proteins in the human body are glycosylated. Protein-bound glycans can be N or O linked and represent the two major types of glycans [91]. Around 700 proteins integrate the glycosylation machinery, making glycan biosynthesis intensively more complex than protein synthesis [92]. Glycosylation is essential for a multitude of biological functions [93] and alterations in glycosylation during the transition from health to disease and disease progression have boosted the scientific interest in studying the glycome [92, 94]. In comparison to other “omics,” however, glycomic databases are still underdeveloped, owning to the complexity of glycan composition, heterogeneity, and the vast variation of branching [95]. In glycomic LC-MALDI-MS, capillary electrophoresis (CE)-ESI-MS and LC-ESI-MS are the most common applied bioanalytical techniques [91]. The glycome could provide a key in understanding the mechanistic links between obesity and metabolic diseases. Recent epidemiological studies suggested that IgG N-glycosylation pattern variations, including lower galactosylation, correlate with measures of obesity and central adiposity [96–99]. IgG galactosylation strongly decreases its proinflammatory activity [96] and its decrease observed in obese individuals could contribute to chronic inflammatory state featured in obesity. Further glycomic research might lead to the discovery of novel inflammatory biomarkers that contribute to obesity development, persistence, and progression.

## Microbiomics

The human microbiome comprises the sum of all human-associated microorganisms (microbiota) living within a well-defined habitat within the human body [100]. Dysbiosis was coined as a term more than 100 years ago to denote the imbalance in the composition and metabolic capacity of the microbiota [101, 102]. Microbiomic studies focus on the better characterization of the microbial structure, function, and composition [100]. Several approaches and analytical techniques have been applied in microbiomics such as sequencing data of the gene that encodes the RNA component of the small ribosomal subunit (16S rRNA) for profiling taxonomic abundance of microorganisms, NGS technologies for metagenomic studies comprising gene identification [100, 103], and microarray-based technologies for meta-transcriptomic analysis [104]. Metabolomic and multi-omics approaches have been also recently applied to study the microbiome function and composition and assess the consequences of host-microbiome interactions [104–106]. Altered gut microbiota composition as measured by a relative increase in the Firmicutes/Bacteroidetes ratio has been commonly reported in obese individuals [107–109]. However, the effect sizes of observed associations of taxonomic composition and obesity in epidemiological studies were generally weak [110] and inconsistent [111]. Beyond taxonomic composition revealed by genomic studies, metagenomic studies reveal information on the genetic functional diversity of the gut microbiota. A recent meta-analysis has shown that gut microbiome metagenomic functional diversity traits and patterns correlate with obesity (e.g., N-glycosylation by oligosaccharyltransferase is depleted in obese individuals), highlighting the importance of the microbiome function over its composition [112]. A recent systematic review comparing concentrations of short-chain fatty acids (SCFA) between obese and lean individuals concluded that obese individuals had higher concentrations of acetate in blood and feces, propionate and valerate in feces, and butyrate in feces produced by fermentation of dietary fiber in the gut. These SCFAs were suggested to play roles in metabolic modulation, appetite regulation, and immune function [113, 114]. Diverse epidemiological studies have shown that exposure to antibiotics in the first year of life is associated with an increased risk of obesity during childhood and adolescence [115–118]. Antibiotic-induced dysbiosis in early life was suggested to lead to obesity development by diverse mechanisms including a decrease of metabolic protective species, by affecting the amount of calories absorbed from the diet, alteration of hepatic function, and hormone secretion, and impaired metabolic signaling [115]. A recent meta-analysis reported a significant dose-response relationship between antibiotic exposure in very early life and childhood adiposity, showing elevated risk with repeated doses [117]. Microbiomic studies also allowed the assessment of probiotics, synbiotics, and prebiotics in the management of obesity. For example, supplementation with synbiotics containing specific strains (e.g., *Lactobacillus gasseri* strains) exerted anti-inflammatory properties and led to efficient weight loss in obese patients [119]. Although research has not succeeded in fully defining the parameters for a health advantageous gut microbiome, studies that emphasize function over composition will be a trend future microbiomic research [120]. Improvement of the knowledge in the interactions between the microbiome and the host health might aid in understanding its etiological role in obesity and in the identification of new targets for precision prevention and therapy.

## “Omics” Biomarkers as Targets for Bariatric Surgery and Weight-Loss Interventions

“Omics” biomarkers have been increasingly explored to assess the effects of weight-loss interventions on the epigenome, transcriptome, metabolome, and microbiome. Bariatric surgery is regarded as the most effective treatment strategy of severe obesity alleviating the risk of obesity-related comorbidities (e.g., type 2 diabetes, cardiovascular diseases) [121]. Changes in “omics” biomarkers could provide additional insight on the pathophysiological mechanisms mediating beneficial effects of weight loss. In this vein, several studies demonstrated significant changes in DNA methylation patterns following bariatric surgery, highlighting the role of the epigenome in mediating beneficial effect of weight loss intervention on metabolic disturbances in obesity [122–124]. Adipose tissue-specific and whole-blood transcriptomic profiles have also shown to be altered after bariatric surgery [121, 123, 125]. Bariatric surgery could especially strongly influence human metabolism captured by metabolomic changes in amino acid, lipids, carbohydrates, or gut microbiome alterations [126]. For example, a small intervention study of 39 morbidly obese patients quantified acylcarnitines, (lyso)phosphatidylcholines, sphingomyelins, amino acids, biogenic amines, and hexoses in serum samples before and 1, 3, and 6 months after bariatric surgery [127]. The findings demonstrated beneficial effects of bariatric surgery on metabolic health by the restoration of the sphingolipid-phospholipid metabolism through the improvement of the lipoprotein profile [127]. Due to the small scale of most bariatric surgery intervention studies, interpretation of complex and extensive data outputs from “omics” data should be done with caution. Meta-analytical and advanced bioinformatic modeling that combine results from multiple intervention studies could provide additional insights on obesity pathophysiology but also evaluate “omics” biomarkers as treatment targets [124].

## Integrative Multi-“omics” and Bioinformatics

The high amount and complexity of data generated by the different high-throughput analytical assays required the development and application of a number of bioinformatic and biostatistic tools to “make sense” of the generated “omics” data [18]. Novel machine learning algorithms, such as deep learning and artificial neural networks, have been gaining popularity as powerful approaches for analysis of heterogeneous and complex data [9]. Advanced bioinformatic methods are especially advantageous in analyzing combinations of “omics” datasets [128]. Integrated multi-“omics” approaches have further emerged as a collective field aiming to obtain a better understanding of the complexity and interactions of the biological systems including those predisposing obesity [129]. Nevertheless, the application of multi-omics approaches has faced a number of challenges including multiple sources of bias arising from differences in study designs, sample collection, measurement, and data analysis methods [128]. Despite the increased availability of analytical and programming options, available bioinformatic approaches bear their own limitations and their application requires further evaluation [130]. Strong epidemiological study design, high laboratory precision and validation, and sound research hypotheses remain fundamental in interpreting integrative “omics”-based analyses along with the integration of multiple “omics” via complex bioinformatic data analysis. Further work is needed to develop analytical infrastructures able to generate, analyze, and interpret multi-“omics” data as a basis for guiding precision prevention strategies.

## Conclusion

Recent technological advances allowed the identification of a number of “omics” biomarkers that brought forward the etiological insights into the mechanisms involved in obesity development. Understanding the role of genetic and epigenetic factors and their influences in the transcriptome, proteome, metabolome, and microbiome became the new frontier in obesity and metabolism research. However, the transformation of large and heterogeneous “omics” data into biological knowledge has proven challenging especially when different methods applied in the same population yield inconsistent results. In this regard, cautious interpretation of findings and further statistical or biological validation of results should represent an important focus of future research. There is an increased demand for larger prospective cohort studies to validate findings and determine biomarker reproducibility before they can find applications in primary care and public health. Despite the current challenges, obesity-related “omics” biomarkers bring substantial promise in identifying new intervention targets and effective public health strategies that pave the way towards patient stratification and precision prevention.
